# Autofluorescence mediated red spherulocyte sorting provides insights into the source of spinochromes in sea urchins

**DOI:** 10.1038/s41598-019-57387-7

**Published:** 2020-01-24

**Authors:** Jonathan Hira, Deanna Wolfson, Aaron John Christian Andersen, Tor Haug, Klara Stensvåg

**Affiliations:** 1 The Norwegian College of Fishery Science, The Faculty of Biosciences, Fisheries and Economics, UiT The Arctic University of Norway, Tromsø, Norway; 2 Department of Physics and Technology, The Faculty of Science and Technology, UiT The Arctic University of Norway, Tromsø, Norway

**Keywords:** Flow cytometry, Cellular imaging

## Abstract

Red spherule cells (RSCs) are considered one of the prime immune cells of sea urchins, but their detailed biological role during immune responses is not well elucidated. Lack of pure populations accounts for one of the major challenges of studying these cells. In this study, we have demonstrated that live RSCs exhibit strong, multi-colour autofluorescence distinct from other coelomocytes, and with the help of fluorescence-activated cell sorting (FACS), a pure population of live RSCs was successfully separated from other coelomocytes in the green sea urchin, *Strongylocentrotus droebachiensis*. This newly developed RSCs isolation method has allowed profiling of the naphthoquinone content in these cells. With the use of ultra high-performance liquid chromatography, UV absorption spectra, and high-resolution tandem mass spectrometry, it was possible to identify sulphated derivatives of spinochrome C, D, E and spinochrome dimers, which suggests that the RSCs may play an important biological role in the biogenesis of naphthoquinone compounds and regulating their bioactivity.

## Introduction

The red spherule cells (RSCs) of sea urchins exhibit intriguing immune responses when the animals are in stressful conditions and during bacterial invasion their concentration within the coelomic fluid escalates^1^, they tend to migrate towards wounds, infections, and tissue grafts^2–4^ and they activate a self-degranulation process that releases bactericidal substances^5–7^. The RSCs are comprised of spherical red pigmented granules^8^. This distinct red pigmentation has been found to be related to the polyhydroxylated naphthoquinone (PHNQ) compounds such as echinochrome A (Ech A), first discovered in *Echinus esculentus*^7^. This compound has been reported to possess antimicrobial^7^, antioxidant and anti-inflammatory^9–11^, and anticardio toxic^12^ properties. A recent study reported that iron-chelating properties of Ech A deter the growth of microorganisms by confiscating iron from the environment^13,14^. In addition to Ech A, a number of other PHNQ metabolites such as spinochromes have been identified in sea urchins^15^. The spinochromes have also been shown to have antimicrobial and antioxidant properties, and have been isolated from the spines, test and coelomocytes of various sea urchins^16–19^. As bioactive compounds, PHNQ metabolites have received much attention as potential therapeutic drugs^15^, for instance, Ech A the active ingredient in the drug Histochrome, which is utilised as antioxidant medication in cardiology and ophthalmology^20^. Recently, the wound healing effect of echinochrome extracted from *Paracentrotus lividus* have also been reported^21^. Exploration of the source of PHNQs and their biogenesis may lead to the discovery of additional novel therapeutic drug lead molecules and a greater understanding of their biological role in sea urchins^15^. Currently, it is unknown whether the RSCs are responsible for producing these PHNQ compounds or not. Hence, in order to perform profiling of the different compounds produced by RSCs, a pure population of RSCs is obligatory.

Several gradient centrifugation methods have been applied in order to separate different coelomocyte types from sea urchins^22^. Such methods have been successfully applied in the separation of phagocytes only (based on their densities), but none of the methods have provided a pure RSCs population^5,13,22^. Fluorescence activated cell sorting (FACS) could be an alternative to facilitate the separation of the desired number of target coelomocytes. Traditionally, FACS requires cell surface antigens to be fluorescently labelled by antibodies for isolating a specific population of live cells^23^. Such an approach provides greater flexibility and a higher yield of a viable population of cells. FACS-based methods can also offer isolation of target cell populations that are already fixed, permeabilized, and labelled with antibodies^23^. It has been shown that, fixed, dissociated specific embryonic cells of (*Strongylocentrotus purpuratus*) could be effectively isolated by FACS and further processed for downstream analysis such as gene expression profiling^24^. However, fixation does not offer the isolation of live cells. The unavailability of antibodies targeting specific cell surface antigens of sea urchin coelomocytes limits the use of FACS based cell separation^24,25^. The results from a recent study indicate that lectin-based FACS could serve as a flow cytometry based strategy to purify distinct purple sea urchin (*S. purpuratus*) coelomocytes^25^. Lectins are known to target glycosylation patterns presented by individual cell types^26–29^ and this interaction could thus be utilised to define different immune cell populations. For instance, peanut agglutinin (PNA), a plant lectin, facilitates the identification of activated germinal centre B cells^30^. The glycosylation pattern usually reflects different cellular states and therefore, may alter lectin interaction. For example, *Solanum tuberosum* lectin (STL) fails to bind phagocytic cells of sea urchins settled onto glass slides, but binds successfully when the cells are in solution^25^. The RSCs of sea urchins are shown to possess a dynamic array of cellular states^31^. It remains elusive how lectin based FACS can be used successfully when lectin interaction may vary depending on the dynamics of RSCs cellular states in response to invading pathogens.

As an alternative to the above-mentioned methods, the autofluorescence exhibited by endogenous molecules^32^ within cells might be utilised for isolating specific cell types. Previous studies have shown that autofluorescence based methods were advantageous over traditional antibody labelling methods in isolating different cell types of both animal and plant origin such as alveolar macrophages^33^, pancreatic islet cells^34^, breast cancer cells^35^, epidermal cells^36^ and plant glandular trichomes cells^37^. Previously, it has been reported that the sea urchins larvae and embryonic pigment cells emanate spectacular autofluorescence^38,39^. A recent study has shown that the RSCs of adult sea urchin emits a weak autofluorescence when excited with 633 nm in the far red channel using FACS and it was suggested that the method requires further optimization for efficient sorting of these cells using FACS^40^.

In this study, we demonstrate a simple and efficient FACS based method to separate and purify live RSCs from *S. droebachiensis* based on their emanated autofluorescence. Additionally, as a downstream analysis, profiling of PHNQ compounds present within the RSCs was performed using ultra-high performance liquid chromatography coupled to a diode array detector and quadrupole time-of-flight mass spectrometer (UHPLC-DAD-MS/MS).

## Results and Discussion

### RSCs exhibit multi-colour autofluorescence

**Figure 1:**
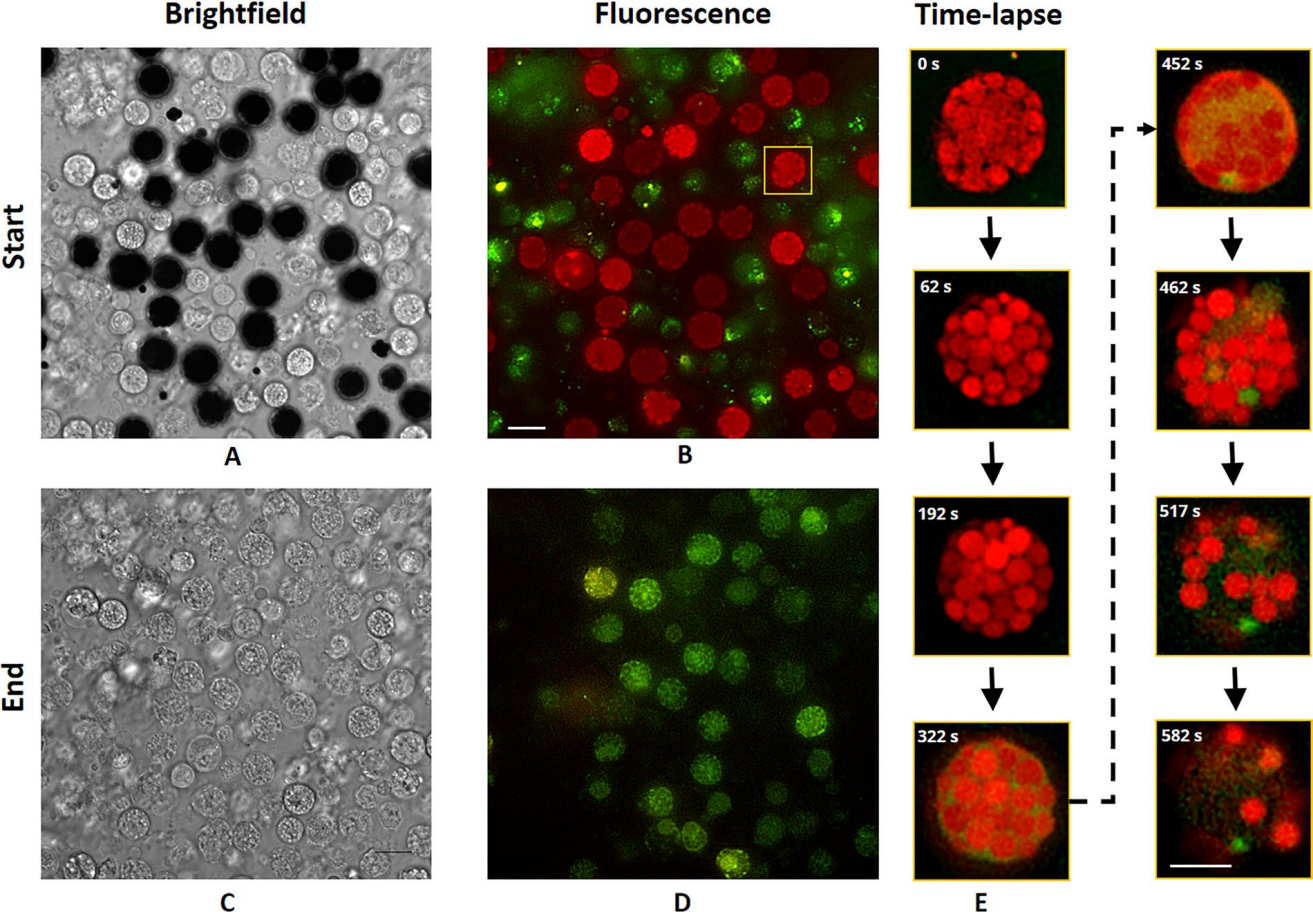
Brightfield and deconvolved fluorescence images of RSCs. (**A**) Bright field and (**B**) fluorescence images of a mix population of CSCs and RSCs at time 0 min. (**C**) Bright field and (**D**) fluorescence images after one hour of continuous fluorescence imaging of the same field of view. (**E**) Close-up fluorescence images at representative time points showing morphological changes in the cell highlighted in (**B**). Scale bar 10 µm (**A–D**) and 5 µm (**E**).

Broad-spectrum autofluorescence was observed in RSCs and recorded using deconvolution microscopy in two channels: orange (ex 543.5/25 and em 593/36 nm) and green (ex 477/30 and em 527/44). To facilitate more accurate sorting of RSCs using FACS, it was important to understand their time-dependent fluorescence properties. In the flow cytometry, each cell is exposed to excitation light for typically only a few microseconds^41^. It is well established that both short and extended light exposure (microseconds to minutes) can change the behaviour of fluorescent molecules, most notably with bleaching (i.e. cessation of the emission of fluorescent light)^42^, but also with shifts of the excitation or emission spectra^43^. Therefore, assessment of the time-dependent fluorescence properties was necessary. From microscopic observation, it was clear that both colours showed distinct spatial distributions within RSCs and time-dependent properties (Fig. 1). Notably, pigmented cells that appeared dark during bright field imaging exhibited significant differences in fluorescence emission than cells that appeared white in bright field (Fig. 1A–D and Vids. S1, 2). The dark cells appear to be RSCs and the colourless white cells are likely colourless spherule cells (CSCs). However, the dark cells from the beginning of an imaging experiment would appear indistinguishable from the white cells at the end of a long time-lapse (Fig. 1C).

As imaging progressed with time-lapse fluorescence assessment, both channels increased in intensity, most notably in the orange channel (Vid. S1). The orange signal appears primarily confined to sac-like compartments, which fill the RSCs, and the green signal appears predominantly in the intracellular space surrounding the sacs. When the orange signal reaches a maximum intensity (typically after ~5–30 minutes of continuous imaging), the spherical sacs quickly begin to disappear in the orange channel, after which the green fluorescence become more visible within the RSCs (Fig. 1B, E). Typically, from a few minutes to over an hour, the intensity in both channels would gradually reduce as well, especially with the orange signal eventually disappearing completely (Fig. 1D). Additionally, correlative brightfield imaging showed that the dark pigment of the RSCs would disappear at the same time as the orange fluorescence would (Vid. S3). After extended imaging, almost all of the cells in a field of view would appear white with bright field imaging, but cells just outside of the illumination area would still retain their dark pigment, indicating that the effect is related to imaging rather than environmental or external causes. Overall, the time-dependent fluorescence assessment summarizes that it takes least several minutes under highly-focused intense illumination for the prominent orange fluorescence to disappear, which suggests that there will be no significant photobleaching effect on the RSCs when they will be excited for only a few microseconds with the light at a wavelength specified for orange fluorescence in the flow cytometry.

Previously it has been reported that the echinochromes show notable optical properties in diethyl ether solution; for example, Ech A appears as an orange-red solution and emitting orange fluorescence^44^. Echinochromes are considered the prominent naphthoquinone compound present in RSCs and we speculate that echinochromes or their PHNQ analogues may have significant roles in multi-colour RSCs autofluorescence. To the best of our knowledge, no echnichromes/spinochromes are commercially available for use as a standard. Thus, isolation and characterization of echinochromes and their analogues, as well as other compounds is crucial to elucidate the cause of the RSCs autofluorescence properties. Hence, profiling the echinochrome types in a pure population of RSCs is an important step prior to identifying and isolating the responsible fluorophore compounds.

### Pure cell population of RSCs

**Figure 2:**
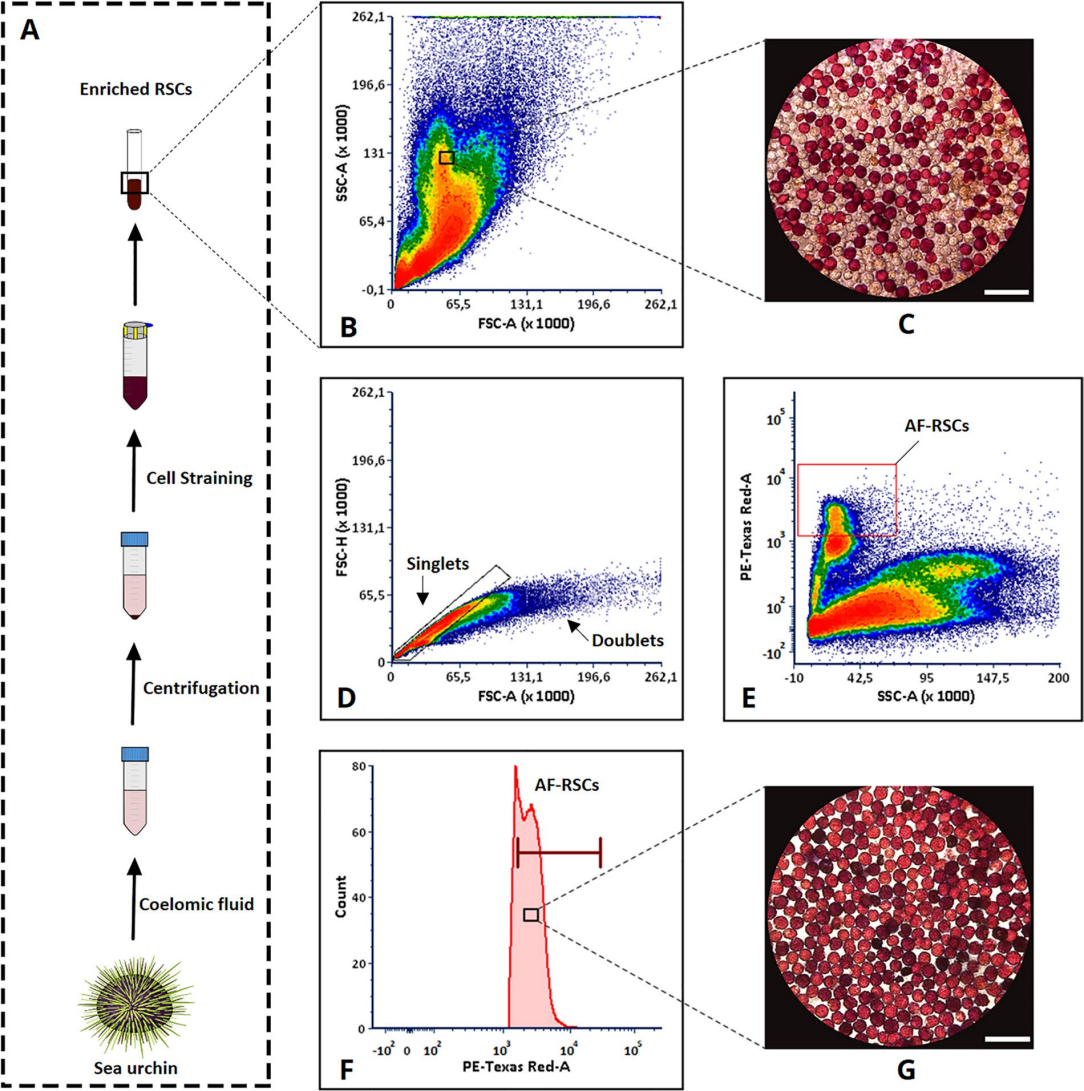
Flow cytometry based autofluorescent (AF) RSCs sorting. Here, FCS Express 6.06.0014 (De Novo Software) generated two-parameter density plot shows the density of cells at a given position represented by different colours. (**A**) Sampling of coelomocytes and RSCs enrichment. The density plot represents; (**B**) Forward/Scatter profile of live unstained coelomocyte. (**C**) Microscopic image of RSCs and other cells before sorting. (**D**) Gating of singlets allowing discrimination of doublets and (**E**) Gating of distinct fluorescent RSCs population represented by the red box. (**F**) Histogram of fluorescent RSCs population. The histogram marker presents the selective population to be sorted. (**G**) Microscopic image of pure AF-RSCs after sorting. Microscopic image scale bar 50 µm.

Following the initial results of the autofluorescence phenomenon exhibited by the RSCs, we speculated that this fluorescence property of RSCs could be utilised as potential discriminating characteristics to isolate these cells as pure populations from other coelomocytes using FACS. We have observed that RSCs emit much stronger orange fluorescence compared to green fluorescence at the initial phase. Therefore, we focused on the orange fluorescence for FACS based cell isolation. The initial investigation using FACS was performed on the enriched RSCs (Fig. 2A), which demonstrated that based on forward scatter (FSC), side scatter (SSC) distribution, the cellular size and granular complexity were insufficient to discriminate RSCs from other coelomocytes, especially CSCs (Fig. 2B,C). This indicates that the basic cell morphology (based on granularity) of the RSCs almost resembles that of CSCs with the exception of the pigmentation properties. To investigate whether the RSCs could be discriminated from the CSCs and other coelomocytes, the enriched RSCs sample was analysed for autofluorescence detection by the PE-TexRed channel in the flow cytometer. The PE-TexRed channel bandpass filter supports the range of excitation and emission wavelength for the orange fluorescence. Prior to defining the specific autofluorescent subpopulation, the doublet cells were excluded to avoid the inaccuracy in single cell sorting using FSC-A (x-axis) versus FSC-H (y-axis) gating strategy (Fig. 2D). After excluding the doublets, the SSC profile (x-axis) and PE-TexRed channel (y-axis) in the two-parameter density plot enabled to detect a well-defined autofluorescent subpopulation, which were further gated for subsequent sorting process (Fig. 2E). Then after establishing a suitable gating parameter, a subpopulation of these autofluorescent cells with high fluorescence signals were targeted for sorting by the flow cytometer as shown in the single parameter fluorescence histogram (Fig. 2F). The autofluorescent cells were then sorted with purity masking employed by the FACS system. With the stringent purity mode, the cells with definite fluorescence signals were sorted accordingly. As a result, the sorted coelomocyte subpopulation were found to be pure (>99%) population of RSCs as demonstrated by microscopic image analysis. Since RSCs are dark-red pigmented cells as observed by eye or white light imaging, it enables them to be distinguished from other coelomocytes, making it convenient to observe the purity of the population under the microscope. After sorting, the RSCs were found devoid of CSCs (Fig. 2G), confirming their purity.

Orange fluorescence observed in the sorted RSCs (using deconvolution fluorescence microscopy) indicates that the cells have an intact cellular morphology (Fig. S1). Concurring with the intact cellular morphology, the sorted RSCs were found to be ~90% viable as determined by their ability to exclude trypan blue dye, which suggested that the sorting and capture medium conditions were suitable for collecting live pure RSCs. The sterile filtered cell-free coelomic fluid (CF-CF) cell capture medium was found suitable for RSCs incubation and favourable for their cellular conditions.

### PHNQ profiling of RSCs

Due to the unavailability of commercial standards, detection of PHNQ features was achieved via UHPLC-DAD-MS/MS based dereplication of known PHNQs (n = 41, Table S1), as well as the analysis of fragmentation spectra, and UV absorption spectra. It was possible to identify several PHNQ-like molecular features within the cell extracts using the combination of these techniques.

**Figure 3:**
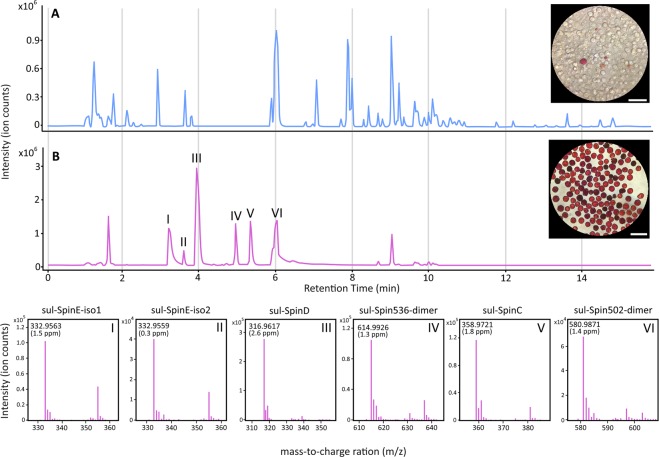
UHPLC-MS analysis of MPCs and RSCs H_2_O extracts. (**A**) Base peak chromatogram of MPCs H_2_O extract. (**B**) Base peak chromatogram of RSCs H_2_O extract depicting the tentative identification of previously unreported sulphate derivatives of spinochromes and suspected previously reported spinochromes. I: Sulphate derivative of spinochrome E isomer 1. II: Sulphate derivative of spinochrome E isomer 2. III: Suspected sulphate derivative of spinochrome D. IV: Suspected sulphate derivative of spinochrome dimer (*m/z* 614.9926). V: Suspected sulphate derivative of spinochrome C. VI: Suspected sulphate derivative of spinochrome related dimer ethylidene-6, 60-bis (2,3,7-trihydroxynaphthazarin) (*m/z* 580.9871). All tentatively identified metabolites were absent in extracts of the MPCs substrate, with the exception of trace amounts of sul-SpinD. Scale bar 50 µm.

Profiling of the mix population of coelomocytes (MPCs) and RSCs H_2_O extracts revealed a much higher proportion of PHNQ compounds in the RSCs extract in comparison to the MPCs extract. An almost complete absence of PHNQ features were observed within the MPCs extract (Fig. 3A), with only a single, low signal-to-noise feature being observed. This is presumed to be the result of a small number of residual RSCs within the MPCs sample (Fig. 3A). In addition to the trace amount of a single PHNQ feature, there was a high abundance of non-PHNQ compounds (lack of high wavelength absorption - Fig. S2) in the MPCs sample. This result significantly contrasted that of the RSCs extract, which predominately featured PHNQ molecular features (Fig. 3B).

A total of 6 PHNQ features were observed in the RSCs extract, demonstrating a high degree of correlation between PHNQ compounds and RSCs, and consequently red autofluorescence. Within the RSCs extract, 2 molecular features (likely structural isomers) were observed that corresponded to the previously reported PHNQ, spinochrome E sulphate (sul-SpinE)^45^. The previous reporting of sul-SpinE noted uncertainty in regards to the moiety conveying the additional 80 u (sulphate or phosphate)^45^. The 2 molecular features detected here can be attributed to the addition of a sulphate due to the mass accuracy of the [M-H]^−^ ion (<2 ppm), as well as the characteristic and accurate mass of the in-source SO_3_ neutral loss fragmentation ion (<2 ppm). In addition to the 2 sul-SpinE molecular features (sul-SpinE-iso1, sul-SpinE-iso2), 4 previously unreported PHNQ features where tentatively identified. These are suspected to be sulphated derivatives of previously reported spinochromes, and were: spinochrome C sulphate (sul-SpinC), spinochrome D sulphate (sul-SpinD), spinochrome 536 dimer sulphate (sul-Spin536-dimer), and spinochrome 502 dimer sulphate (sul-Spin502-dimer) (Fig. 3B). All detected PHNQ molecular features were found exclusively within the RSCs extract, with the exception of trace amounts of sul-SpinD within the MPCs extract.

In addition to accurate mass and isotope spacings, fragmentation patterns of the [M-SO_3_-H]^−^ ions (Figs. S2–6) were used to support the tentative identification of these metabolites. The fragmentation patterns of sul-SpinC, sul-SpinD, and sul-SpinE revealed a series of neural losses of CO, characteristic of substituted naphthoquinones^46^. In addition, the fragmentation of sul-SpinC resulted in a unique and prominent neutral loss of C_2_H_4_O, possibly resulting from an acetyl moiety, a functional group present in spinochrome C, but absent in spinochromes D and E. The fragmentation of the [M-SO_3_-H]^−^ ions of the dimers resulted in two complementary fragmentation ions, specifically, for sul-Spin536-dimer, *m/z* 237.0045 and *m/z* 297.0259, and for sul-Spin502-dimer, *m/z* 237.0043 and *m/z* 263.0197. The [M-SO_3_-H]^−^ ions were selected for fragmentation due to significant in-source fragmentation of the pseudo-molecular ions resulting in a higher abundance of the SO_3_ neutral loss fragmentation ion. Finally, UV absorption spectra were also obtained which indicated all metabolites constitute significant conjugated systems (Figs. S2–7), similar to those in related spinochromes^47^, with a notable absorption maxima in the range of 502–506 nm.

This large proportion PHNQ sulphate esters and the absence of the de-sulphated analogues is unprecedented in extracts of Echinoidea^15,45^. The typical extraction procedure for these metabolites involves very low pH conditions to dissolve the calcium carbonate portions (usually the tests/spines) of the organism^16,18,19,48^. It is known that sulphate esters can undergo hydrolysis in low pH conditions, and aryl sulphates are particularly susceptible to hydrolysis^49^. Thus, the low pH conditions of the typical acid extraction procedures may lead to the hydrolysis of the PHNQ sulphate esters. This may also explain the original detection of sulphate derivatives, as the first report of sulphated PHNQ utilised formic acid^45^, a weak acid, rather than the typical strong acid, HCl. The use of formic acid may have resulted in a slower rate of hydrolysis, resulting to the initial detection of sulphate derivatives. The hydrolysis of the spinochrome sulphate esters under typical extraction procedures (concentrated HCl ethyl acetate extraction) was investigated and detailed in supporting text (Text S1).

Sulphate esters are common in marine natural products (sulphate esters within Marinlit marine natural products database: n = 1098)^50^. A number of studies have demonstrated various changes in biological activities of natural products due to variations in the number of sulphate esters^51–57^. At least one study comparing a class of marine natural products demonstrated a correlation between sulphate groups and reduced biological activity^49^, representing a detoxifying effect. Other studies have also demonstrated significant differences between sulphated and de-sulphated analogues of natural products, with many showing a decrease in biological activity for sulphate analogues^53–55^. Although a decrease in activity is common for sulphated derivatives, one study has described an increase in biological activity^57^, and another described an increase in activity for a disulphated analogue compared to its monosulphated counterpart^52^. It has also been shown that the addition of a sulphate ester can reverse a biological activity, changing a metabolite from a receptor agonist to an antagonist^51^. These changes in activity make the sulphate derivatives of spinochromes an interesting targeted for further biological investigation. Importantly, extraction of these spinochromes as pure compound will be beneficial for understanding their role in the autofluorescence properties of RSCs.

## Conclusions

The present findings confirm that RSCs exhibit a distinct multi-colour autofluorescence, which undoubtedly has enabled the efficient isolation of pure live RSCs populations from sea urchins using FACS. We anticipate the isolation of pure live RSCs population using this method will be valuable for in-depth transcriptomic studies to provide a better understanding on the biological function of RSCs and their immune responses. Additionally, based on our results we speculate that RSCs may be a major source of the PHNQ compounds in sea urchins. *In vitro* culture studies of RSCs will therefore be beneficial for understanding the biogenesis pathway of spinochromes and other bioactive compounds.

## Methods

### Ethical statement

The present study includes experimental procedures performed in compliance with the national and international ethical guidelines as well guidelines from UiT The Arctic University of Norway. Importantly, animals (sea urchin) used in this study do not belong to any group of endangered species and any ethical restrictions to use for research purpose. The animals were sacrificed immediately after sampling of coelomic fluid.

### Chemicals

Liquid chromatography mass spectrometry (LC-MS) grade H_2_O, acetonitrile (ACN) and formic acid for UHPLC-DAD-MS/MS analysis and HPLC grade methanol (MeOH), reagent grade diethyl ether, hydrochloric acid (HCl, 37%), and NaCl used for sample preparation were all purchased from VWR Chemicals (Oslo, Norway). Milli-Q H_2_O (Merck; Darmstadt, Germany) was used in sample and buffer preparation. Purine and HP-0921, Agilent part number: G1969-85003, were used as internal standards for MS spectra calibration, purchased from Matriks AS (Oslo, Norway). In this study, calcium and magnesium (Ca^2+^/Mg^2+^) free seawater (CMFSW)^58^ and calcium and magnesium free modified anticoagulant buffer containing 462 mM NaCl, 10.7 mM KCl, 7 mM Na_2_SO_4_, 2.1 mM NaHCO_3_, and 20 mM EGTA (CMFSW-E), pH 7.8^59^ were used.

### Animals

Live green sea urchins, *Strongylocentrotus droebachiensis* (Müller, 1776), were collected off the coast of Tromsø, Norway. The animals were maintained healthy in a large tank equipped with unaltered circulating seawater at 6–8 °C under dark/light conditions. The animals were regularly fed with artificial sea urchin feed (kindly provided by Nofima, Tromsø, Norway) until the sample collection.

### Coelomocyte preparation

To obtain coelomic fluid from the sea urchins, syringes attached with 22 G 2” needles were inserted through the peristomial membrane around the animal´s mouth. Immediately after drawing the coelomic fluid, it was mixed with an equal volume of ice-cold calcium and magnesium (Ca^2+^/Mg^2+^) free anticoagulant buffer (CMFSW-E). The additional coelomic fluid was drawn without adding CMFSW-E and CF-CF was prepared by centrifuging this (800 × g, 20 min, 4 °C). The clot inhibited coelomic fluid (CI-CF) was centrifuged (150 × g, 2 min, 4 °C), allowing both colourless spherule cells (CSCs) and RSCs to sediment^59^. After centrifugation, the resulting supernatant was separated and the pellet was resuspended in a CF-CF. The supernatant represents the mix population of coelomocytes (MPCs) comprising a higher percentage (>90%) of non-RSCs types (phagocytes, vibratile cells, and few CSCs). The MPCs were later prepared and subjected for LC-MS analysis. The resuspended pellet enriched with both CSCs and RSCs, was passed through a cell straining (100 µm and 20 µm) process in order to remove any tissue debris and clumps/aggregates of cells. The resulting highly concentrated single cell suspension of mixed spherule cells was then subjected to fluorescence microscopy and FACS.

### Fluorescence imaging and image processing

Fluorescence imaging was performed on a DeltaVision Elite deconvolution microscope (GE Healthcare), which captures widefield fluorescence images and then applies a deconvolution algorithm to improve the resolution and contrast. The system is equipped with a 60 × 1.42NA oil immersion objective and a sCMOS camera. Fluorescence images were acquired with the fluorescein isothiocyanate (FITC) and tetramethylrhodamine (TRITC) filter sets with excitation from 477/30 nm and 543.5/25 nm, respectively, and emission of 527/44 nm and 593/36 nm, respectively. Time-lapse images were acquired approximately every 2.6 seconds. Fluorescence images were first deconvolved using Softworx, which was provided by the microscope manufacturer. Image analysis, including contrast adjustment, was performed using Fiji software (https://fiji.sc).

### RSCs sorting

To sort the RSCs, the BD FACSAria III instrument (BD Biosciences) was configured suitably for RSCs. The instrument was pre-calibrated with BD CST beads and the fluidic startup was performed using the in house prepared sterile sheath fluid (CMFSW, pH 7.6, mOsm 940 ± 2). Drop delay was adjusted with BD AccuDrop beads (BD Biosciences) and the temperature inside the sample injection chamber was set to 4 °C. To analyse the cell size and granular complexity, the channel voltages were set as follows: Forward scatter (FSC, 130 V), side scatter (SSC, 346 V). Cellular autofluorescence was measured by the PE-TexRed channel (bandpass filter excitation, 561 nm yellow-green laser; emission, 610/20 nm; 530 V) using a neutral density (ND) filter 1.5. Autofluorescent cells were distinguished from other cell types by their distinct PE-TexRed values (y-axis) against SSC values (x-axis) subsequently after performing doublet discrimination based on FSC-A, area (x-axis) and FSC- H, height (y-axis). Based on the autofluorescence signals, the gate was defined by BD FACSDiva™ software 8.0.1 (BD Biosciences) and pure subpopulation of RSCs were sorted. The sorting was performed with a 70 μm nozzle with a sheath pressure of 70 psi. Freshly sorted cells were collected in sterile filtered (0.1 μm) CF-CF supplemented with 10 mM CaCl_2_ and 10 mM HEPES as cell-capture media. For the viability assessment, the sorted cells were collected in CMFSW supplemented with 10 mM HEPES (CMFSW-H). The viability of the sorted cells was estimated by trypan-blue exclusion (0.2% v/v, final concentration) using FastRead counting slides. As a post-sort analysis, the cell population purity was confirmed by microscopic (Zeiss Axiovert 40 CFL) method. Colour images of pure RSCs populations were captured with iPhone 6 s connected to an ocular lens (10 x) with an adapter and a macro zoom lens (14 x, olloclip). The captured images were then processed with Cytosketch (Cytocode), Fiji, and Photoshop CS6 (Adobe). The sorting and validation of pure RSCs population were performed in several replicates over different time-periods to assess the reproducibility of the RSCs sorting method.

### LC-MS sample preparation

Both MPCs and pure RSCs population (>99%) were subjected to a H_2_O based extraction procedure. After enriching the RSCs population, the resulting supernatant (~40 ml coelomic fluid) were centrifuged at 3500 × g, 4 min, 4 °C and the resulted MPCs pellets were subjected for further analysis. Additionally, the RSCs (~1 × 10^6^ cells) isolated by FACS were also centrifuged at 3500 × g, 4 min, 4 °C. Next, 1.5 ml of H_2_O (ratio ~1.5: 40 of H_2_O to original coelomic fluid volume) was added to each resulting MPCs and RSCs pellets. This mixture was placed on a vortex mixer for 10 s, followed by centrifugation (12000 × g, 5 min). The supernatants were collected and immediately submitted for UHPLC-DAD-MS/MS analysis.

For comparison to typical extraction procedures used on Echinoidea^16–19,48^, HCl (485 µL) was added to a sub-sample of the RSCs H_2_O extract (500 µL) to make a final concentration of 6 M HCl and left at room temperature for 1 h. This sample was then partitioned with diethyl ether (1 mL) three times, and the diethyl ether phases were pooled. The organic phase was then washed by partitioning with 5% NaCl solution (3 mL) three times. The diethyl ether phase was then dried under a nitrogen stream at 30 °C, re-dissolved in 50% MeOH (400 µL), centrifuged (12000 × g, 4 min), and submitted for UHPLC-DAD-MS analysis.

### Liquid chromatography and mass spectrometry

Analysis of both MPCs and RSCs extracts were undertaken by UHPLC-DAD-MS/MS. Chromatographic separation was achieved with the use of an Agilent 1290 Infinity UHPLC (Matriks; Oslo, Norway) equipped with a Phenomenex Kinetex F5 UHPLC column (1.7 µm, 100 Å, 150 × 2.1 mm) (Værløse, Danmark) utilizing gradient elution. The gradient consisted of two eluents, eluent A consisting of H_2_O and eluent B consisting of ACN, each containing 0.1% formic acid. Gradient elution began with 5% eluent B, increased to 100% B over 15 min, held at 100% B for 2 min, at a constant flow rate of 0.3 mL·min^−1^ and a constant column temperature of 40 °C. Injection volume was 10 µL. The UHPLC was coupled to an Agilent 1290 Infinity Diode Array Detector (DAD), set to detect absorption between 190 and 600 nm. The DAD was further coupled to an Agilent 6540 QTOF MS. Ionization was achieved using an electrospray ionization source (ESI), with analysis undertaken in negative polarity mode. Drying gas temperature was set to 350 °C, drying gas flow rate was 13 L·min^−1^, nebuliser pressure was 35 psi, and the capillary voltage was 4000 V. Mass spectra were obtained at *m/z* 100–1700 at a rate of 3 scans·s^−1^ in the standard acquisition, and 4 scans·s^−1^ in tandem MS. In tandem MS analysis, compound specific collision energies were used to obtain optimal fragmentation of suspected PHNQ molecular features. Fragmentation spectra were obtained at *m/z* 50–1500. All MS analysis was undertaken with internal standards for spectra calibration: purine and HP-0921.

Data analysis was performed using Qualitative Analysis B.07.00 SP2, and dereplication was performed using the Find by Molecular Formula function, using a target list comprised of known PHNQ secondary metabolites from the class Echinoidea (Table S1). Due to the lack of commercially available reference standards, tentative identification of metabolites was aided with the use of fragmentation analysis in comparison to literature fragmentation patterns of substituted naphthoquinones and dimers^46,60^ as well as UV absorption spectra.

## Supplementary information

Supplementary information

.Supplementary information 2

Supplementary information 3

Supplementary information 4
