# Supplementary material for: Autofluorescence mediated red spherulocyte sorting provides insights into the source of spinochromes in sea urchins
Source: Sci Rep. 2020 Jan 24;10:1149. doi: 10.1038/s41598-019-57387-7 (PMC6981155; doi:10.1038/s41598-019-57387-7)
Supplement: Supplementary file 1 — Supplementary information [file 41598_2019_57387_MOESM1_ESM.pdf]

SUPPLEMENTARY MATERIAL

Autofluorescence mediated red spherulocyte sorting provides insights into the source of spinochromes in sea urchins

Authors: Jonathan Hira [1,*], Deanna Wolfson [2], Aaron John Christian Andersen [1], Tor Haug [1] and Klara Stensvåg [1,*]

[1] *The Norwegian College of Fishery Science, The Faculty of Biosciences, Fisheries and Economics, UiT The Arctic University of Norway, Tromsø, Norway*

[2] *Affiliation Department of Physics and Technology, The Faculty of Science and Technology, UiT The Arctic University of Norway, Tromsø, Norway*

***** Correspondence: klara.stensvag@uit.no and jonathan.hira@uit.no

Text S1

Analysis of the HCl treated, diethyl ether extracted, H_2O extract of the RSCs revealed an absence of the sulphated analogues, and the appearance of molecular features corresponding to their de-sulphated counterparts. Each of these de-sulphated derivatives had no detectable indication of the in-source fragmentation of SO_3, each had increased retention times (relative to their sulphate ester counterpart), indicating a decrease in polarity (Fig. S8). Furthermore, a change in UV absorption spectre (Fig. S9), support the conclusion that these are indeed de-sulphated derivatives. These results show that low pH conditions typically utilised in Echinoidea extraction procedures have the potential to hydrolyse the sulphate esters of PHNQ, and may explain the absence or low abundance of sulphated derivatives seen in previous studies. It is likely, that the mild H_2O extraction produce utilised for the RSCs may preserved the metabolite profile of these cells. Preserving these constituents for analysis may provide insight into the function of these metabolites in vivo, and is necessary to assess their biological activity.

Videos

Video S1: Timelapse fluorescence depiction of MPCs and RSCs population.

Video S2: Timelapse fluorescence depiction of single RSCs.

Video S3: Timelapse fluorescence and brightfield depiction of RSCs.

Figures

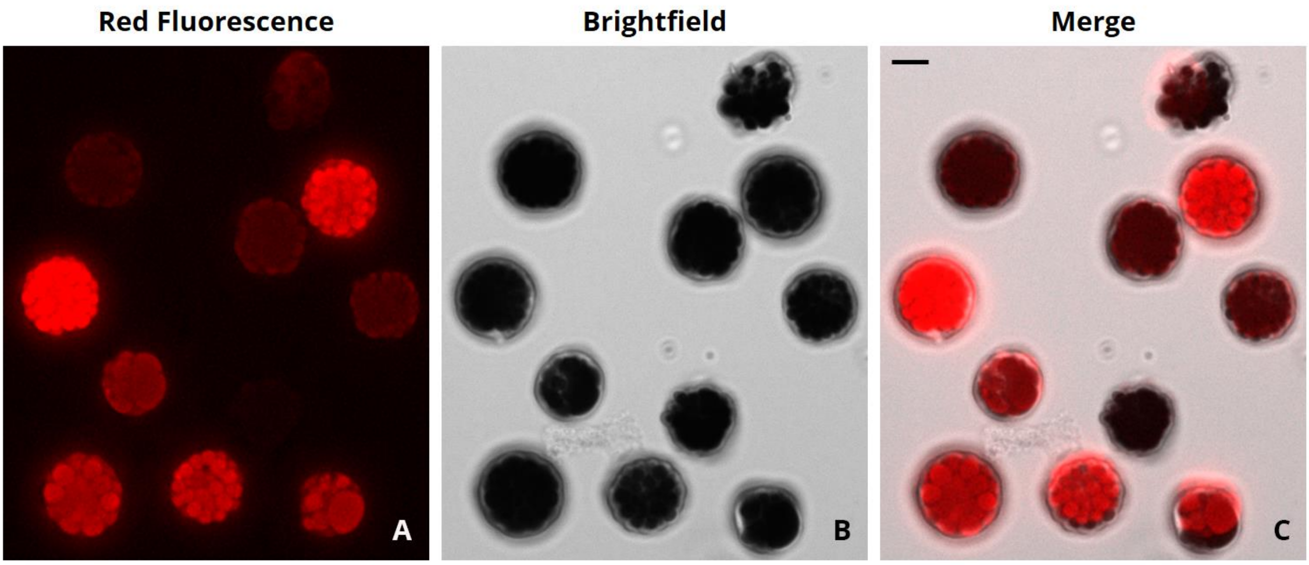

Figure S1: Fluorescence image of pure RSCs population.

(A) Orange fluorescence image. (B) Brightfield image and (C) Merged between fluorescence and brightfield image. Scale bar 5 μm.

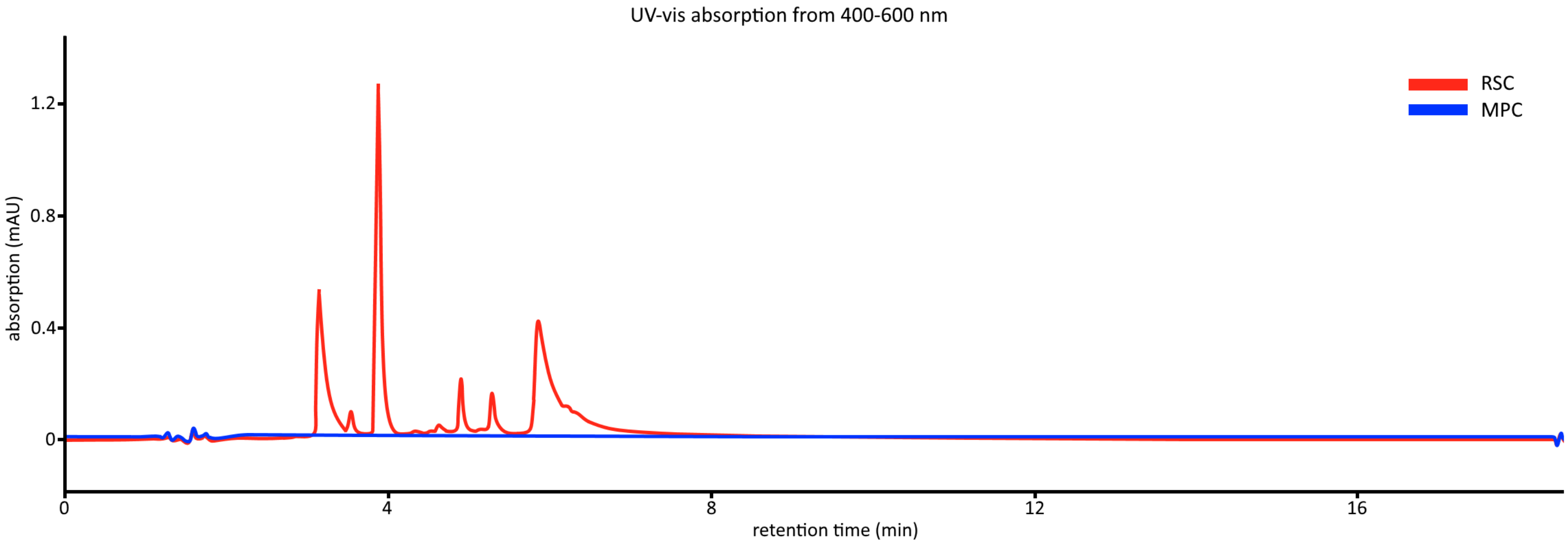

Figure S2: UV-vis absorption chromatogram at 400-600 nm for RSCs (red), and MPCs (blue). Chromatogram of high wavelength absorption, showing the difference between the absorption of the RSCs extract and the MPCs extract at 400-600 nm, range which includes characteristic absorption maxima of PHNQ.

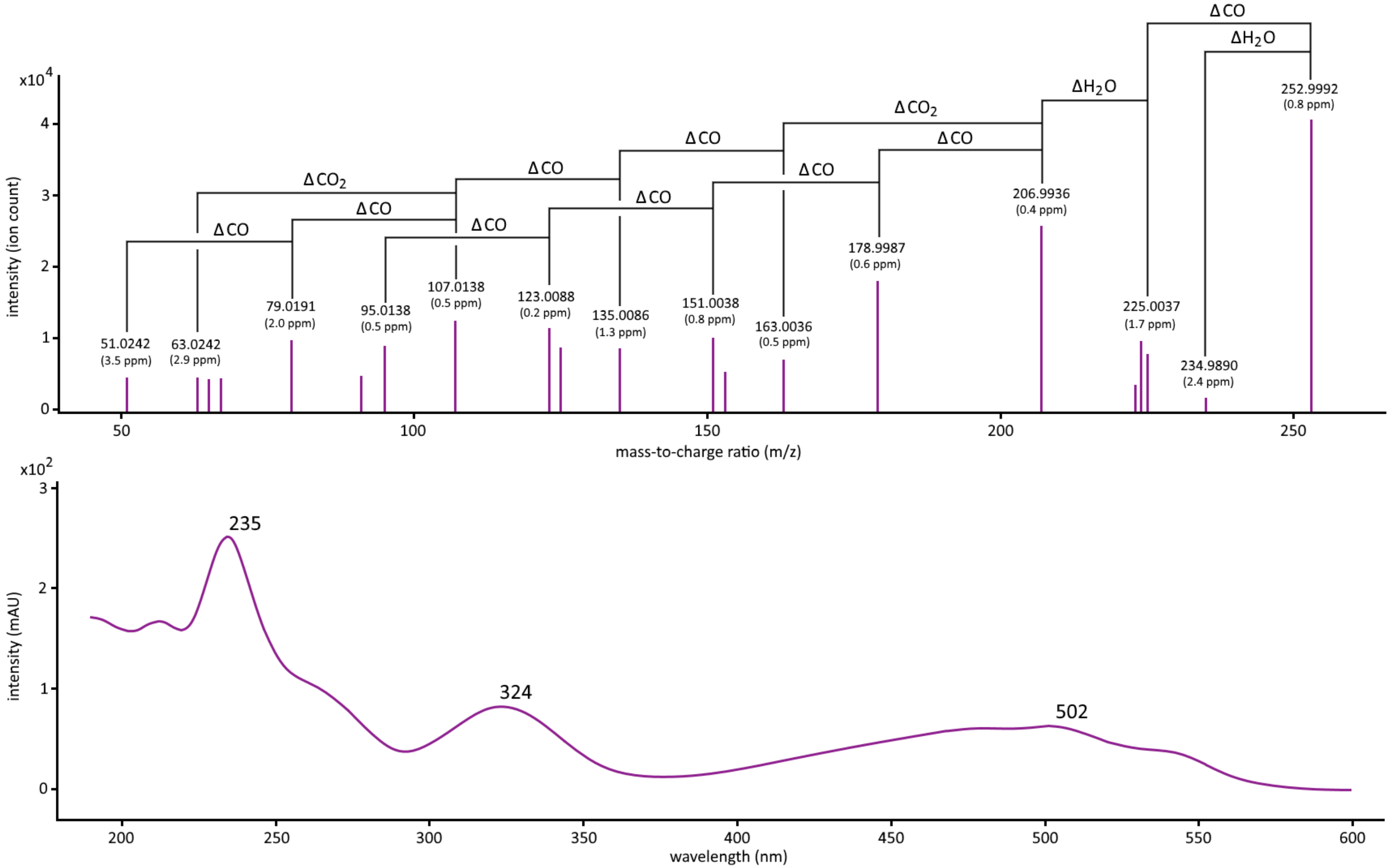

Figure S3: Fragmentation pattern and UV-vis absorption spectrum of tentatively identified spinochrome E sulphate iso 1. Fragmention of the [M-SO_3-H]$^-$ ion *m/z* 252.9990 with a CID energy of 25 CeV. Approximate eluent: 25% ACN 0.1% formic acid.

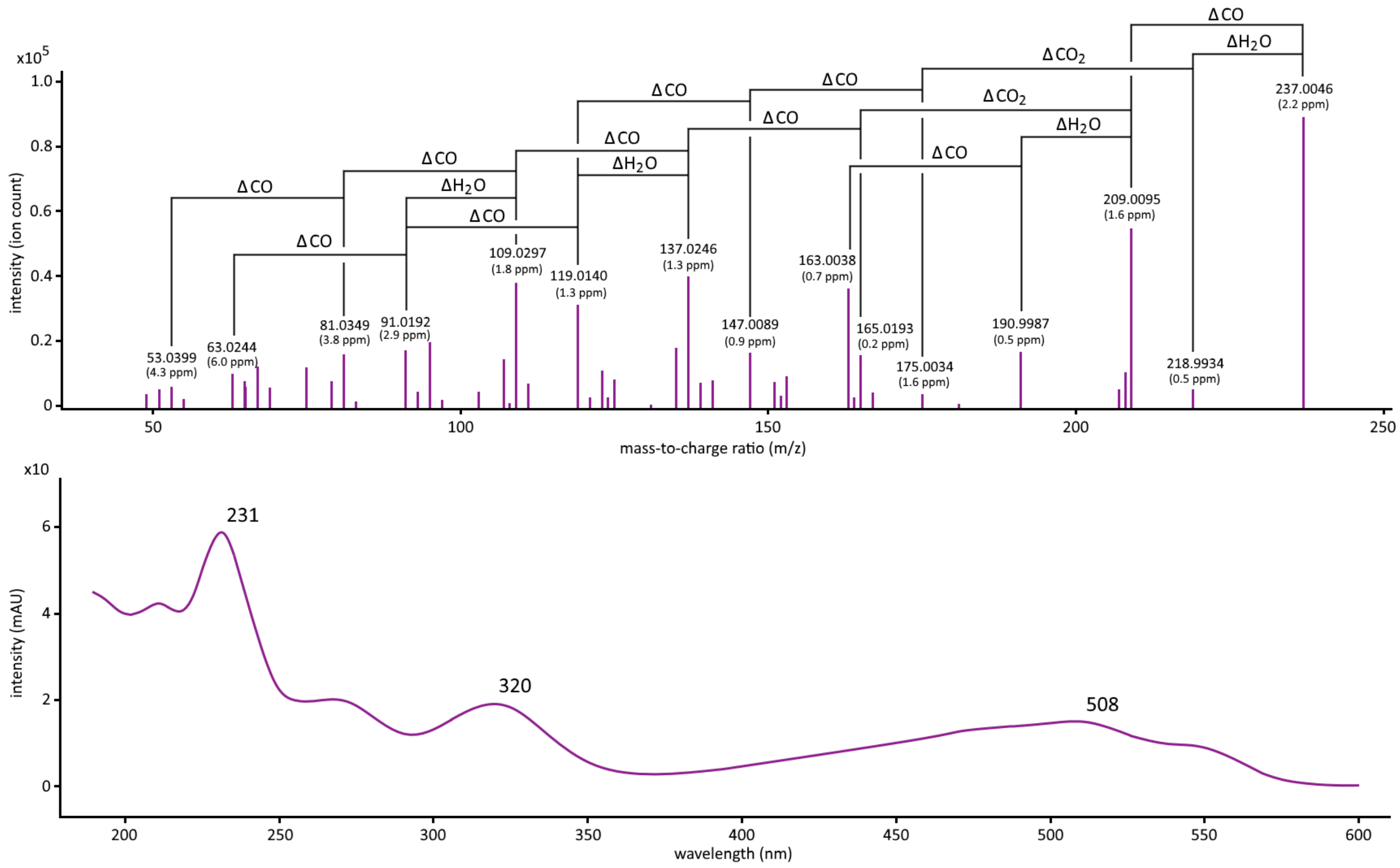

Figure S4: Fragmentation pattern and UV-vis absorption spectrum of suspected spinochrome D sulphate. Fragmention of the $[M\text{-}SO_3\text{-}H]^-$ ion *m/z* 237.0041 with a CID energy of 25 CeV. Approximate eluent: 30% ACN 0.1% formic acid.

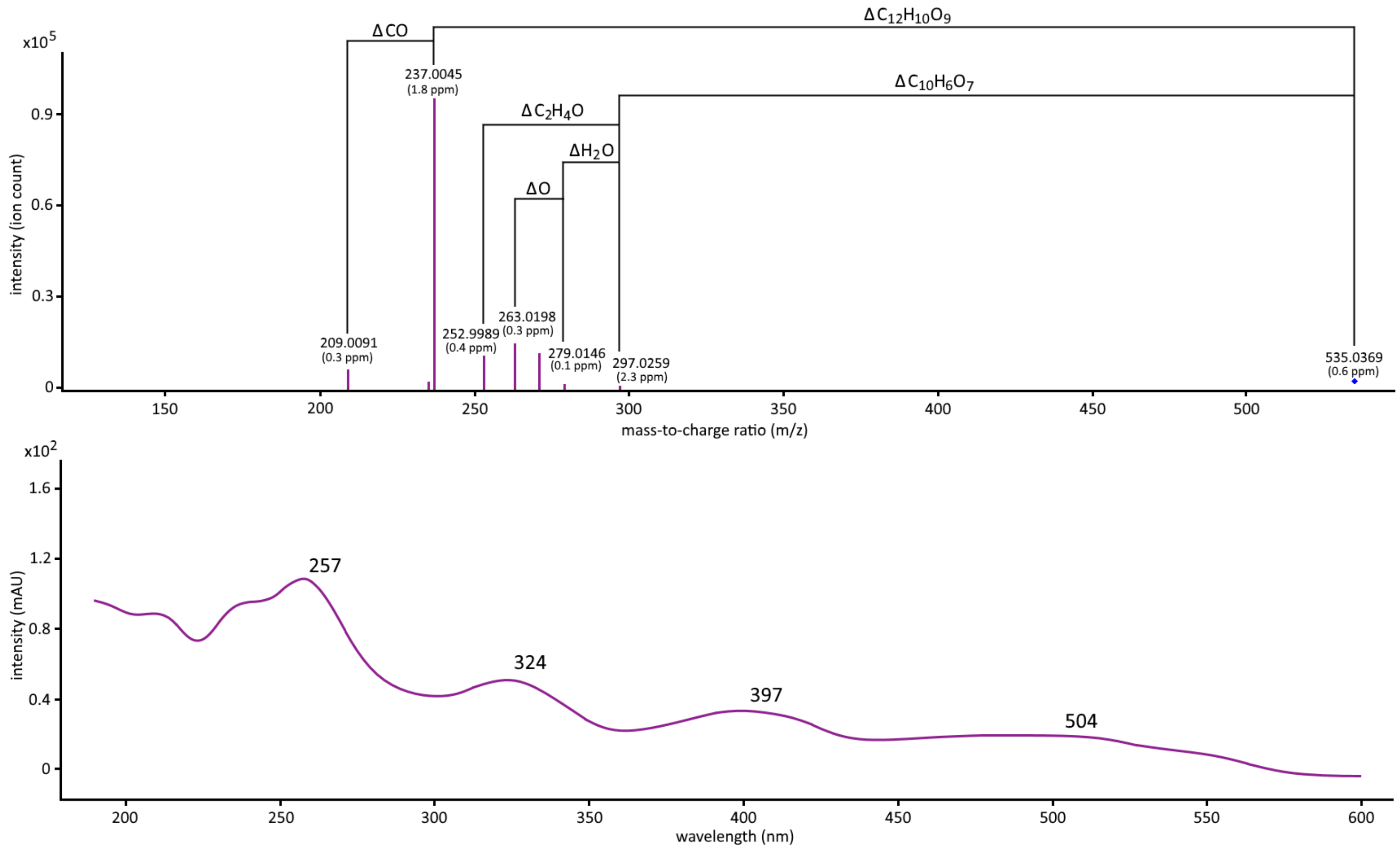

Figure S5: Fragmentation pattern and UV-vis absorption spectrum of suspected spinochrome 536 dimer sulphate. Fragmention of the $[M\text{-}SO_3\text{-}H]^-$ ion *m/z* 535.0366 with a CID energy of 25 CeV. Approximate eluent: 38% ACN 0.1% formic acid.

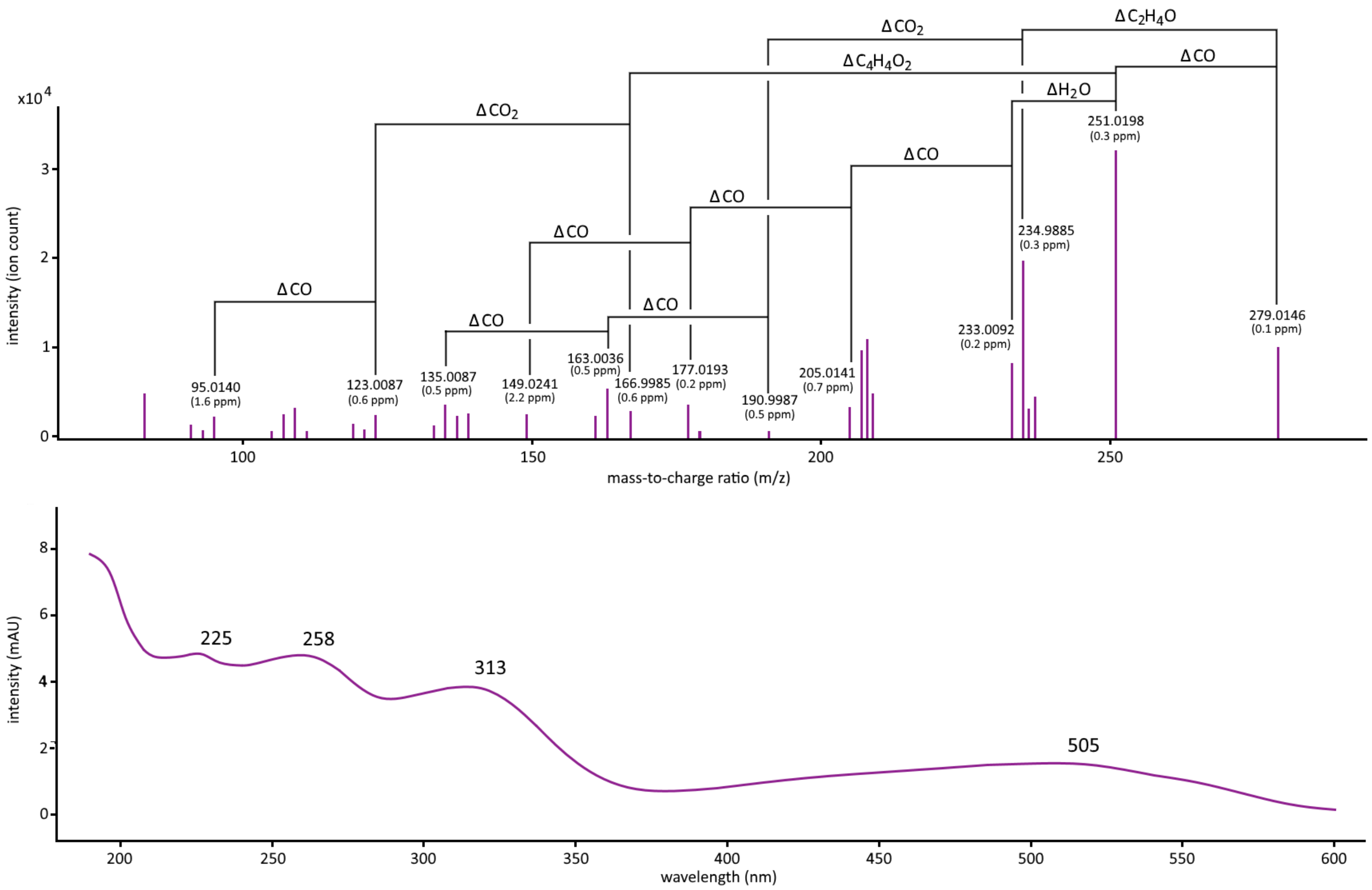

Figure S6: Fragmentation pattern and UV-vis absorption spectrum of suspected spinochrome C sulphate. Fragmention of the [M-SO_3-H]$^-$ ion *m/z* 279.0146 with a CID energy of 30 CeV. Approximate eluent: 40% ACN 0.1% formic acid.

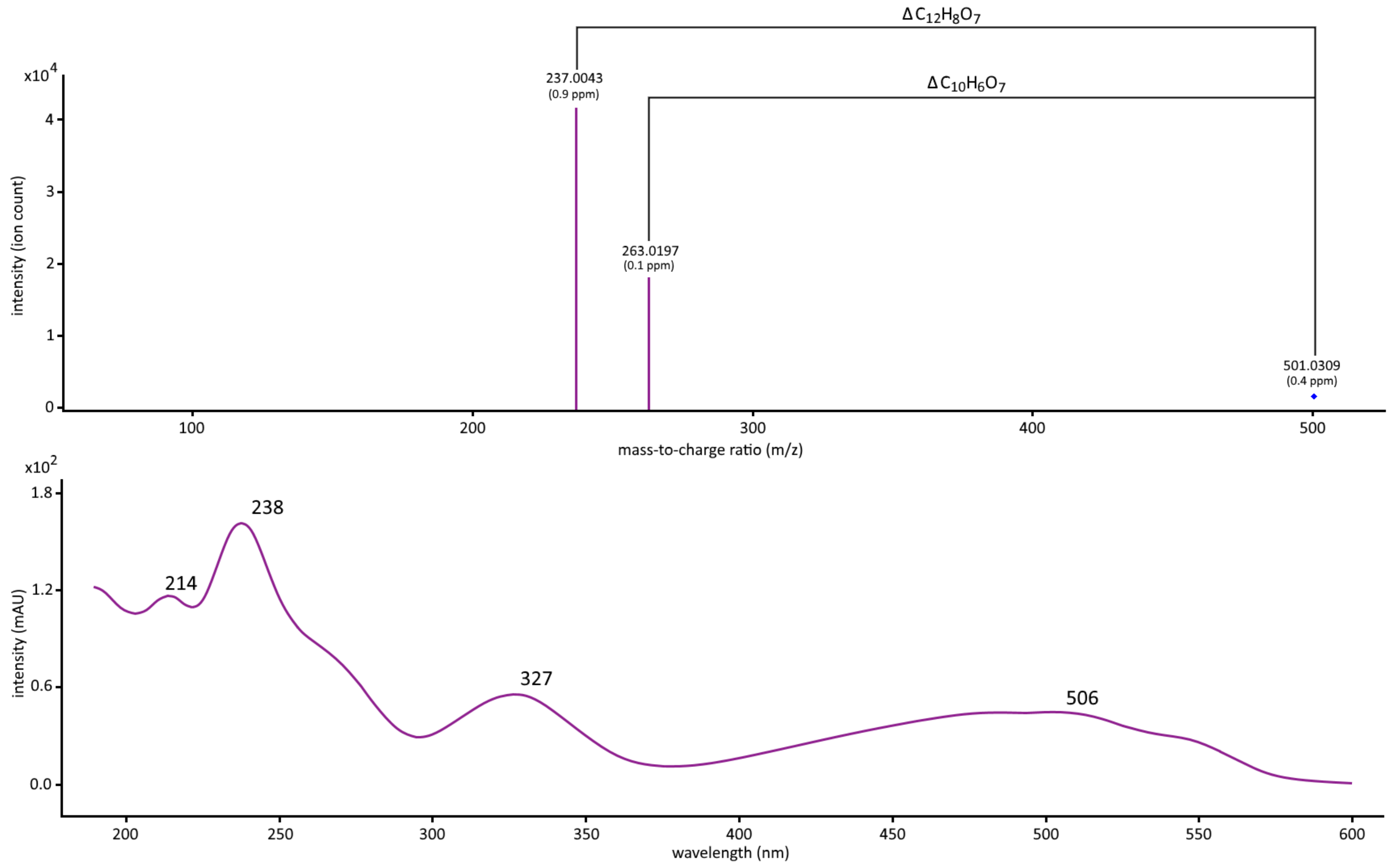

Figure S7: Fragmentation pattern and UV-vis absorption spectrum of suspected spinochrome 502 dimer sulphate. Fragmention of the $[M\text{-}SO_3\text{-}H]^-$ ion *m/z* 501.0311 with a CID energy of 25 CeV. Approximate eluent: 45% ACN 0.1% formic acid.

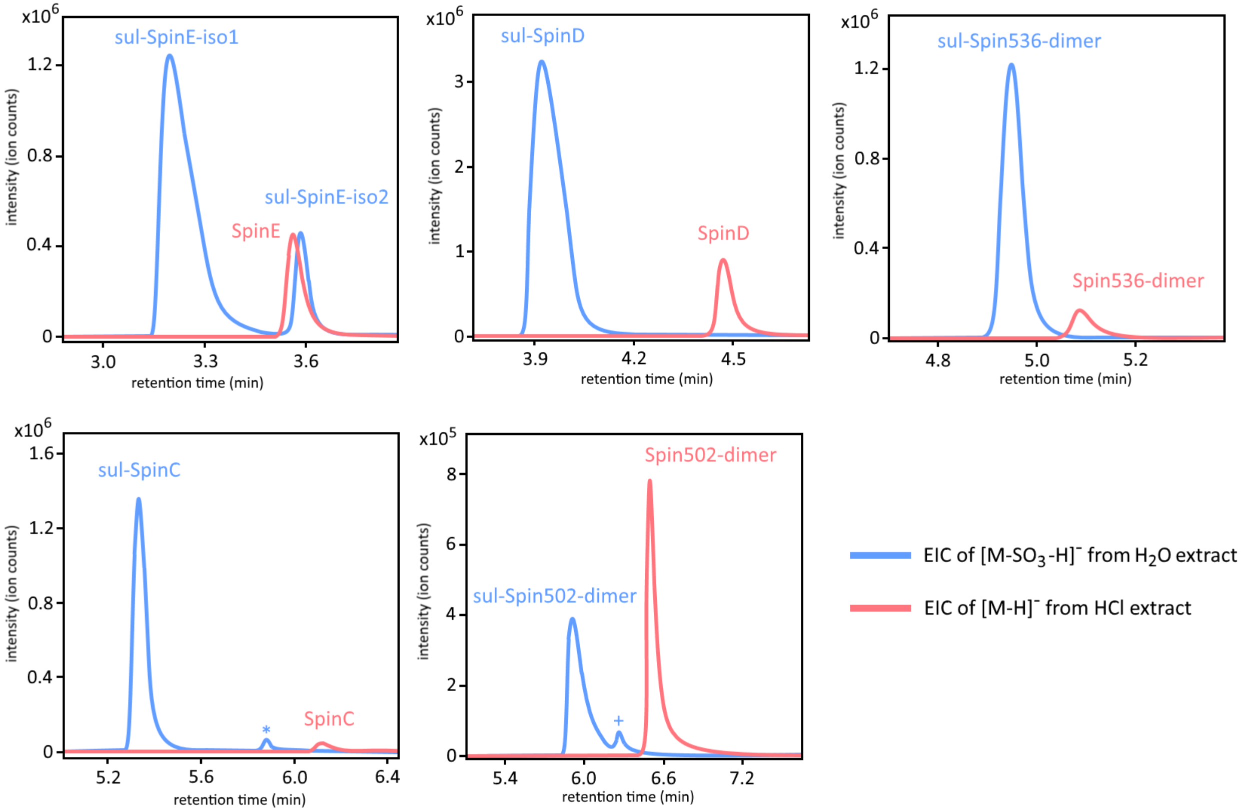

Figure S8: Comparison of retention times of suspected spinochrome sulphate derivatives to that of de-sulphated forms. Extracted ion chromatograms of $[M\text{-}SO_3\text{-}H]^-$ ions in water extract in blue, and extracted ion chromatograms of $[M\text{-}H]^-$ ions in acidic extract in red. +: potential additional minor isomer of sulphated spinochrome 502 dimer. *: potential additional minor isomer of sulphated spinochrome C.

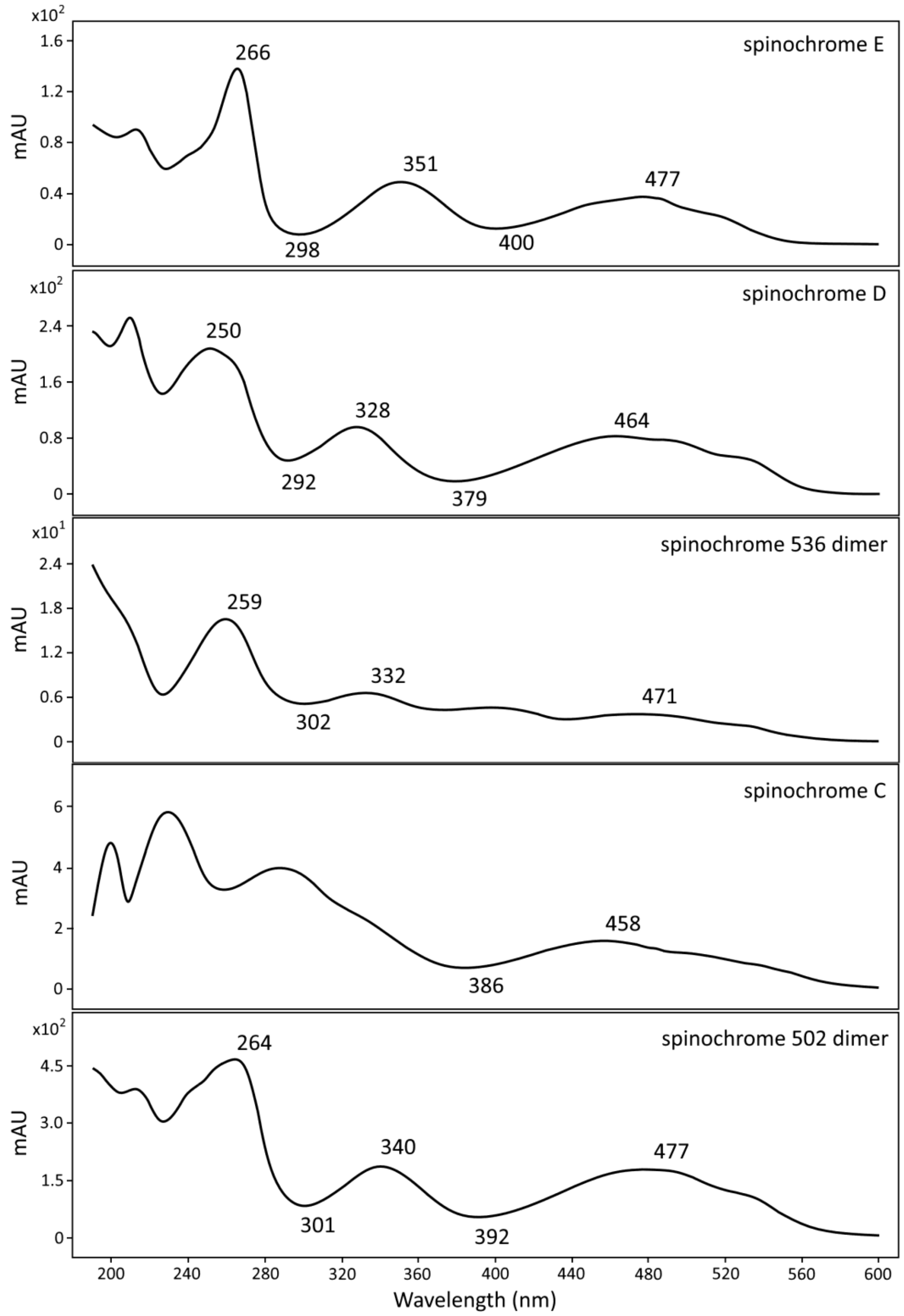

Figure S9: UV-vis absorption spectrum of tentatively identified de-sulphated spinochromes. UV-vis absorption spectrum of tentatively identified de-suphated spinochromes from acidic extracted cells. Approximate eluent (top to bottom) in 0.1% formic acid: 30% ACN; 35% ACN; 40% ACN; 46% ACN; 49% ACN.

Table S1: Target list of known spinochrome-like metabolites used for the purpose of LC-MS dereplication.

#	Compound name	Molecular formula	Exact mass	Genus	Species
1	Echinochrome A	$C_{12}H_{10}O_7$	266.04266	Arbacia	stellata
2	Spinochrome A	$C_{12}H_8O_7$	264.02701	Echinus	esculentus
3	Spinochrome B	$C_{10}H_6O_6$	222.01643	Paracentrotus	lividus
4	Spinochrome C	$C_{12}H_8O_8$	280.0187	Paracentrotus	lividus
5	Spinochrome D	$C_{10}H_6O_7$	238.01135	Mesocentrotus	nudus
6	Spinochrome E	$C_{10}H_6O_8$	254.00627	Mesocentrotus	nudus
7	Spinochrome G	$C_{12}H_8O_7$	264.02701	Echinothrix	diadema
8	Spinochrome S	$C_{12}H_8O_7$	264.02701	Salmacis	sphaeroides
9	2-Hydroxy-3-acetylnaphthazarin	$C_{12}H_8O_6$	248.03209	Echinothrix	diadema
10	2,3,7-trihydroxy-6-ethyljuglone	$C_{12}H_{10}O_6$	250.04775	Echinothrix	diadema
11	6-Ethyl-2-hydroxynaphthazarin	$C_{12}H_{10}O_4$	218.05791	Echinothrix	diadema
12	Naphthopurpurin	$C_{10}H_6O_5$	206.02153	Echinothrix	diadema
13	6-Ethyl-2-hydroxynaphthazarin	$C_{12}H_{10}O_5$	234.05283	Echinothrix	calamaris
14	6-Acetyl-2,7-dihydroxyjuglone	$C_{12}H_8O_6$	248.03209	Echinothrix	diadema
15	6-Acetyl-2-hydroxynaphthazarin	$C_{12}H_8O_6$	248.03209	Echinothrix	diadema
16	Mompain (2,7-Dihydroxy-naphthazarin)	$C_{10}H_6O_6$	222.01643	Echinothrix	diadema
17	Ethylmompain (3-Ethyl-2,7-dihydroxynaphthazarin)	$C_{12}H_{10}O_6$	250.04775	Echinothrix	diadema
18	6-Ethyl-3,7-dihydroxy-2-methoxy-naphthazarin	$C_{13}H_{12}O_7$	280.0583	Diadema	antillarum
19	6-Ethyl-2,7-dihydroxy-3-methoxy-naphthazarin	$C_{13}H_{12}O_7$	280.0583	Diadema	antillarum
20	3-Acetyl-2,7-dihydroxy-6-methylnaphthazarin	$C_{13}H_{10}O_7$	278.04266	Mesocentrotus	nudus
21	Echinamine A	$C_{12}H_{11}O_6N$	265.0586	Scaphechinus	mirabilis
22	Echinamine B	$C_{12}H_{11}O_6N$	265.0586	Scaphechinus	mirabilis
23	Aminopentahydroxynaphthoquinone	$C_{10}H_6O_7N$	253.0145	Mesocentrotus	nudus
24	Spinamine E	$C_{10}H_6O_7N$	253.015	Strongylocentrotus	pallidus
25	Spinazarin	$C_{10}H_6O_6$	222.01643	Scaphechinus	mirabilis
26	Ethylspinazarin	$C_{12}H_{10}O_6$	250.04775	Scaphechinus	mirabilis
27	Tetrahydroxydimethoxynaphthoquinone	$C_{12}H_{10}O_8$	282.03757	Strongylocentrotus	polyacanthus
28	Namakochrome	$C_{11}H_8O_8$	268.02192	Strongylocentrotus	droebachiensis
29	Ethylidene-6,60-bis(2,3,7-trihydroxynaphthazarin)	$C_{22}H_{14}O_{14}$	502.03836	Strongylocentrotus	intermedius
30	Ethylidene-3,30-bis(2,6,7-trihydroxynaphthazarin)	$C_{22}H_{14}O_{14}$	502.03836	Spatangus	purpureus
31	Anhydroethylidene-6,60-bis(2,3,7-trihydroxynaphthazarin)	$C_{22}H_{12}O_{13}$	484.0203	Strongylocentrotus	droebachiensis
32	Anhydroethylidene-3,30-bis(2,6,7-trihydroxynaphthazarin)	$C_{22}H_{11}O_{13}$	484.0203	Spatangus	purpureus
33	Mirabiquinone A (7,50Anhydro-ethylidene-6,60-bis(2,3,7-trihydroxynaphthazarin))	$C_{22}H_{12}O_{13}$	484.0205	Scaphechinus	mirabilis
34	Pyranonaphthazarin	$C_{14}H_{10}O_6$	274.04774	Echinothrix	diadema
35	Acetylaminotrihydroxynaphthoquinone	$C_{10}H_9O_6N$	263.0352	Mesocentrotus	nudus
36	Spinochrome dimer	$C_{22}H_{16}O_{16}$	536.0359	Strongylocentrotus	droebachiensis
37	Spinochrome B sulfate derivative	$C_{10}H_5O_9S$	300.96543	Psammechinus	miliaris
38	Spinochrome E sulfate derivative	$C_{10}H_5O_{11}S$	333.9553	Psammechinus	miliaris
39	Spinochrome A—Iso 2	$C_{12}H_8O_7$	264.027	Toxopneustes	pileolus
40	Spinochrome D—Iso 1	$C_{10}H_6O_7$	238.01135	Diadema	savignyi
41	Spinochrome D—Iso 3	$C_{10}H_6O_7$	238.01135	Tripneustes	gratilla
